# Infectious Disease Physicians’ Knowledge and Practices Regarding Wastewater Surveillance, United States, 2024

**DOI:** 10.3201/eid3010.240719

**Published:** 2024-10

**Authors:** Carly Adams, Libby Horter, Susan E. Beekmann, Philip M. Polgreen, Jessica N. Ricaldi, Souci Louis, Scott Santibañez

**Affiliations:** Centers for Disease Control and Prevention, Atlanta, Georgia, USA (C. Adams, L. Horter, J.N. Ricaldi, S. Louis, S. Santibañez);; Goldbelt Professional Services, Chesapeake, Virginia, USA (L. Horter);; University of Iowa, Iowa City, Iowa, USA (S.E. Beekmann, P.M. Polgreen)

**Keywords:** Wastewater surveillance, wastewater, Centers for Disease Control and Prevention, infectious diseases, physicians, survey, pathogens, bacteria, viruses, fungi, parasites, antimicrobial resistance, United States

## Abstract

A survey of US infectious disease physicians indicated that few regularly reviewed wastewater surveillance (WWS) data but many reported examples of how WWS has affected or could affect their clinical practice. WWS data can be useful for physicians, but increased communication between public health professionals and physicians regarding WWS could improve its utility.

Although clinical reporting is critical to infectious disease surveillance, it is limited to the interaction of individual patients with the healthcare system. Wastewater surveillance (WWS) has a history of detecting disease early, independent of healthcare-seeking behavior or access to healthcare and testing (*1*). WWS data often correlate with transmission levels found in case-based surveillance and can strengthen efforts to prevent disease transmission (*1*). To enhance the capacity to detect SARS-CoV-2 and additional microbial and chemical targets in wastewater, the US Centers for Disease Control and Prevention (CDC; Atlanta, GA, USA) established the National Wastewater Surveillance System (https://www.cdc.gov/nwss/wastewater-surveillance.html) during the COVID-19 pandemic (*2*). As of April 2024, a total of 1,690 NWSS sites in all 50 states and several cities and tribal communities have been monitoring wastewater for infectious diseases. Data on SARS-CoV-2 and monkeypox virus are publicly available (*3*–*6*).

Often, the first to diagnose and report infectious diseases are physicians (*7*). However, before physicians’ initial interactions with case-patients, public health and clinical awareness of infections can be enhanced by WWS. To describe the knowledge and practices of US infectious disease physicians regarding WWS, we surveyed the Emerging Infections Network (EIN), a provider-based network supported by CDC and the Infectious Diseases Society of America (*8*). The activity was reviewed by CDC and conducted consistent with applicable federal law and CDC policy (e.g., 45 C.F.R. part 46, 21 C.F.R. part 56; 42 U.S.C. §241(d); 5 U.S.C. §552a; 44 U.S.C. §3501 et seq.).

During February–March 2024, we distributed a 9-question cross-sectional survey to EIN members (Appendix). We described the survey responses received from 448 (25%) of 1,809 US-based infectious disease physician members and summarized them by respondent characteristics. We identified example quotations that summarized themes identified most frequently from the open-ended question.

Although 64% of respondents knew of WWS in their county or state of work, 36% reported uncertainty or no WWS occurrence (Tables 1, 2). Respondents in the midwestern and western United States were more aware of WWS than those in the northeastern and southern United States (Appendix Table 1). A total of 22% respondents reviewed WWS data regularly, 56% did not review regularly, and 22% were not aware of those data (Table 3). For data sources, 43% of respondents used CDC websites (*3**–**6*) and 28% used non-CDC websites. Among the 108 respondents who listed non-CDC websites, most reported state health department websites (58%), followed by local health department and private/academic institution websites (25% each). Targets considered most useful for WWS included influenza A (67%), influenza B (57%), respiratory syncytial virus (55%), norovirus (55%), and measles (55%). Providers with <15 years of experience were more likely to consider measles a useful target than were providers with ≥15 years of experience (Appendix Table 2). Facility-level reporting was most often considered very useful for hospitals (60%) and long-term care facilities (54%). Compared with providers of adult healthcare, providers of pediatric healthcare were less likely to consider reporting for long-term care facilities very useful and were more likely to consider reporting for kindergarten–12th grade schools very useful (Appendix Table 3).

**Table 1:** Characteristics of 448 infectious disease specialists responding to Emerging Infections Network survey regarding wastewater surveillance, February–March 2024*

Respondent practice characteristics	No. (%) respondents
Experience since infectious disease fellowship, y	
<5	63 (14)
5–14	132 (30)
15–24	92 (21)
>25	161 (36)
Practice type	
Adult	356 (80)
Pediatric	92 (21)
Type of hospital in which respondent primarily practices	
City/county	25 (6)
Community	94 (21)
Nonuniversity teaching	106 (24)
University	195 (44)
Outpatient only	3 (1)
US Department of Veterans Affairs or Department of Defense	25 (6)
US Census Bureau region in which respondent resides	
Northeast	108 (24)
Midwest	107 (24)
South	126 (28)
West	107 (24)

*The Infections Disease Society of America Emerging Infections Network is a provider-based emerging infections sentinel network established in 1995 to assist the Centers for Disease Control and Prevention and other public health authorities with surveillance for emerging infectious diseases and related phenomena. The electronic survey was distributed via 3 email messages in February and March 2024 to all US Emerging Infections Network infectious disease physician members.

**Table 2:** Perspectives of 448 infectious disease specialists regarding wastewater surveillance, Emerging Infections Network survey responses, February–March 2024*

Survey responses	No. (%) respondents
Wastewater surveillance conducted in county or state of work	
Yes	286 (64)
No	18 (4)
Unsure	144 (32)
Awareness and frequency of review of wastewater surveillance data, n = 446	
Not aware of those data	97 (22)
Aware but do not review regularly	251 (56)
Aware and review regularly	98 (22)
Use CDC wastewater websites, n = 446	
Yes	194 (43)
No	252 (57)
Type of CDC wastewater websites used, n = 44†	
COVID WVAL map	150 (34)
COVID data tracker	142 (32)
COVID variants	74 (17)
Mpox	37 (8)
Use non-CDC wastewater websites, n = 440	
Yes	125 (28)
No	315 (72)
Targets respondents chose as potentially useful for wastewater surveillance‡	
Influenza A	302 (67)
Influenza B	254 (57)
Respiratory syncytial virus	247 (55)
Norovirus	245 (55)
Measles	245 (55)
Antimicrobial resistance	198 (44)
West Nile virus	172 (38)
Fungal	138 (31)
Bacterial	117 (26)
Adenovirus	110 (25)
Parasitic diseases	103 (23)
Dengue virus	100 (22)
Reported geographic reporting level as very useful‡§	
City, n = 424	281 (66)
County, n = 431	277 (64)
State, n = 426	103 (24)
Nation, n = 418	64 (15)
Reported facility reporting level as very useful‡§	
Hospital, n = 426	254 (60)
Long-term care facility, n = 423	230 (54)
K-12 school, n = 414	151 (37)
University, n = 408	121 (30)
Jail/detention center, n = 414	129 (31)
Homeless shelter, n = 410	138 (34)

*The Infections Disease Society of America Emerging Infections Network is a provider-based emerging infections sentinel network established in 1995 to assist the Centers for Disease Control and Prevention and other public health authorities with surveillance for emerging infectious diseases and related phenomena. The electronic survey was distributed via 3 email messages in February and March 2024 to all US EIN infectious disease physician members. n values indicate no. respondents when <448. CDC, Centers for Disease Control and Prevention; K–12, kindergarten through 12th grade; WVAL, Wastewater viral activity levels. †CDC COVID WVAL Map (https://www.cdc.gov/nwss/rv/COVID19-currentlevels.html); CDC COVID Data Tracker (https://covid.cdc.gov/covid-data-tracker/#wastewater-surveillance); CDC COVID variants (https://www.cdc.gov/nwss/rv/COVID19-variants.html); CDC Mpox (https://www.cdc.gov/nwss/wastewater-surveillance/mpox-data.html); non-CDC wastewater websites (e.g., from state/local health departments or private/academic organizations). ‡At the time of the survey, the National Wastewater Surveillance System was reporting wastewater surveillance data publicly for SARS-CoV-2 and monkeypox virus at the sampling site, state, and national levels. §Respondents were asked “In your practice, please indicate the usefulness of the following potential levels of reporting...” and to rate geographic location and facility reporting levels for wastewater surveillance data as slightly, somewhat, or very useful.

**Table 3:** Thematic summary of specific examples provided by infectious disease physicians to open-ended question of how wastewater surveillance has affected or could affect their clinical practice, Emerging Infections Network, February–March 2024*

Themes identified and subset of example responses, respondents providing free-text responses, n = 192	No. (%) respondents
Situational awareness	91 (47)
“In the absence of reliable Covid test reporting, we integrate wastewater with emergency department and admission data to assess risk.”	
“Identify potential outbreaks early.”	
“I look to tell immunocompromised patients’ risk for COVID.”	
“It has helped with discussions with my patients and their families.”	
“Raises or lowers my clinical suspicion based on prevalence.”	
“Potential early indicator of disease activity in the community.”	
“Include the information in communicable diseases reports for my organization and county.”	
IPC decisions	47 (24)
“Wastewater surveillance has helped us direct IPC and public health resources and can be very helpful.”	
“We have used this information to support decisions to determine need for staff masking during respiratory virus season.”	
“We are using it to inform masking policy and to a lesser extent visitation policy at our hospital.”	
“Providing hospital personal protective equipment guidance.”	
“For many COVID protocols particularly when de-implementing, such as when we were planning to discontinue pre procedure and inpatient admissions screening, this was one of the metrics we used.”	
Diagnostic testing and differential diagnoses	29 (15)
“Increases/decreases suspicion for certain infections prior to results of testing.”	
“Determining which respiratory virus panel is worth offering.”	
“Knowing something is increasing before clinically evident has helped inform testing in our emergency department.”	
“Affects decision to retest for COVID in patients with consistent symptoms with a negative initial test, who might be eligible for remdesivir or Paxlovid.”	
Vaccinations	24 (13)
“Serves as educational and clinical tools to emphasize need for vaccination.”	
“To encourage flu, mpox, and COVID vaccinations when the uptick is seen in the community.”	
“Influenza activity and initiation of vaccination schedules.”	
Healthcare preparedness	19 (10)
“Predicting COVID needs”.	
“Hospital surge planning and resource allocation.”	
“Used for staffing.”	
“During the past few years, I used it as a leading indicator to help with hospital planning as part of my role as Medical Director for Infection Control in my Hospital System.”	
Clinical management	18 (9)
“Increasing resistance genes in our area may lower my threshold to use broader empiric antibiotics.”	
“Incidence of resistant organisms can guide therapy.”	
“Management of vulnerable patient populations such as transplant recipients and other immunocompromised.”	
“Lower thresholds towards treatment.”	
Variant/emerging infections detection	8 (4)
“Watching for SARS-CoV-2 variant emergence.”	
, “Perhaps detection of new strains of influenza.”	
“Best use would be for novel pathogens of interest or antibiotic resistance in specific facilities.”	
No effect	14 (7)
“No impact.”	
“Unless there is practical and evidenced based guidance that is tied to wastewater surveillance results, I do not believe this will be useful for my clinical practice.”	

*The Infections Disease Society of America Emerging Infections Network is a provider-based emerging infections sentinel network established in 1995 to assist the Centers for Disease Control and Prevention and other public health authorities with surveillance for emerging infectious diseases and related phenomena. The electronic survey was distributed via 3 email messages in February and March 2024 to all US EIN infectious disease physician members. IPC, infection prevention and control.

In 192 free-text responses, 178 (40%) of the 448 respondents reported how WWS has affected or could affect their clinical practice and 14 respondents reported that WWS does not affect their clinical practice (Table 3). The most common (47%) response was improving situational awareness, including advanced warning of surges and outbreaks to guide patient counseling. For example, a respondent noted, “If we see measles suddenly popping up in a community, we know we have to act.” Respondents reported using WWS to guide healthcare infection prevention and control decisions (24%) and diagnostic testing and differential diagnoses (15%). When prompted for additional comments regarding WWS, 47 respondents replied. Providers described the utility of WWS (n = 10), how WWS is not useful (n = 5), and the need to better understand correlations with clinical disease (n = 4).

Results from our survey may not be generalizable to all US infectious disease physicians; respondents may have a greater interest in public health surveillance than nonparticipating physicians. However, respondents’ practice characteristics were comparable to those of all EIN members (Appendix Figure). It is unknown how nonrespondents would have answered. If nonresponse resulted from lack of awareness or review of wastewater data, our findings underestimate the need for increased communication with physicians regarding WWS.

Among our findings, many respondents reported examples of how wastewater data affected or could affect their clinical practice, but few reviewed wastewater data regularly. Many respondents were not aware of WWS in their county or state; however, WWS is currently conducted in all 50 states. Increased messaging about the public availability of WWS data is needed. In addition, endemic respiratory viruses, including influenza A, were most commonly reported as useful pathogens for WWS. That finding is consistent with another national survey of infectious disease subject matter experts (*9*). Last, local WWS data were reported as most useful. Currently, CDC reports wastewater data publicly at the sampling site, state, and national levels. Our survey revealed that increased communication between public health professionals and physicians regarding WWS, along with more local reporting, could increase WWS utility.

Appendix Additional information for study of infectious disease physicians’ knowledge and practices regarding wastewater surveillance, USA, 2024.

## References

[1] Kilaru P, Hill D, Anderson K, Collins MB, Green H, Kmush BL, et al. Wastewater surveillance for infectious disease: a systematic review. Am J Epidemiol. 2023;192:305–22. 10.1093/aje/kwac175 36227259 PMC9620728

[2] Adams C, Bias M, Welsh RM, Webb J, Reese H, Delgado S, et al. The National Wastewater Surveillance System (NWSS): from inception to widespread coverage, 2020-2022, United States. Sci Total Environ. 2024;924:171566. 10.1016/j.scitotenv.2024.171566 38461979 PMC11103741

[3] Centers for Disease Control and Prevention. COVID data tracker: wastewater surveillance. 2024 Apr 8 [cited 2024 Apr 8]. https://covid.cdc.gov/covid-data-tracker/#wastewater-surveillance

[4] Centers for Disease Control and Prevention, National Wastewater Surveillance System. COVID-19 current wastewater viral activity levels map. 2024 Apr 4 [cited 2024 Apr 8]. https://www.cdc.gov/nwss/rv/COVID19-currentlevels.html

[5] Centers for Disease Control and Prevention, National Wastewater Surveillance System. COVID-19 variants in wastewater. 2024 Apr 4 [cited 2024 Apr 8]. https://www.cdc.gov/nwss/rv/COVID19-variants.html

[6] Centers for Disease Control and Prevention. National Wastewater Surveillance System. U.S. mpox wastewater data. 2024 Apr 3 [cited 2024 Apr 8]. https://www.cdc.gov/nwss/wastewater-surveillance/mpox-data.html

[7] Thacker SB, Choi K, Brachman PS. The surveillance of infectious diseases. JAMA. 1983;249:1181–5. 10.1001/jama.1983.03330330059036 6823080

[8] Pillai SK, Beekmann SE, Santibanez S, Polgreen PM. The Infectious Diseases Society of America Emerging Infections Network: bridging the gap between clinical infectious diseases and public health. Clin Infect Dis. 2014;58:991–6. 10.1093/cid/cit932 24403542 PMC4634883

[9] Sheth K, Hopkins L, Domakonda K, Stadler L, Ensor KB, Johnson CD, et al. Wastewater target pathogens of public health importance for expanded sampling, Houston, Texas, USA. Emerg Infect Dis. 2024;30:14–7. 10.3201/eid3008.231564 39043434 PMC11286076

