# Supplementary material for: Infectious Disease Physicians’ Knowledge and Practices Regarding Wastewater Surveillance, United States, 2024
Source: Emerg Infect Dis. 2024 Oct;30(10):2217–20. doi: 10.3201/eid3010.240719 (PMC11431906; doi:10.3201/eid3010.240719)
Supplement: Appendix — Additional information for study of infectious disease physicians’ knowledge and practices regarding wastewater surveillance, USA, 2024. [file 24-0719-Techapp-s1.pdf]

Article DOI: https://doi.org/10.3201/eid3010.240719

EID cannot ensure accessibility for supplementary materials supplied by authors. Readers who have difficulty accessing supplementary content should contact the authors for assistance.

Infectious Disease Physicians' Knowledge and Practices Regarding Wastewater Surveillance, USA, 2024

Appendix

Survey Distribution and Questions

US infectious disease physician members of the EIN, which also includes other infectious disease healthcare professionals with advanced degrees (e.g., PharmD, nurse practitioner, and physician assistant), public health members, physicians no longer practicing clinical medicine, and international members, were emailed a link to the electronic survey on February 27, 2024. Completion of the survey was voluntary. Recipients received two email reminders (on March 5, 2024 and March 13, 2024) before the survey closed on March 18, 2024.

EMERGING INFECTIONS NETWORK QUERY

Clinical Utility of Wastewater Surveillance

1. To your knowledge, is wastewater surveillance being conducted anywhere in the county or state where you work?

__ Yes __ No __ Not sure

2. Please rate your current awareness and frequency of review of wastewater surveillance data:

__ I am aware and review these data regularly

__ I am aware but do not review regularly

__ I am not aware of these data

3. Do you use CDC wastewater Web sites? *[Select all that apply]*

__ Yes, COVID-19 Current Wastewater Viral Activity Levels Map

__ Yes, COVID Data Tracker

__ Yes, COVID-19 Variants in Wastewater

__ Yes, U.S. Mpox Wastewater Data

__ No

Any additional information about how you use these Web sites would be very helpful:

4. Do you use other, non-CDC wastewater surveillance Web sites (e.g., from a state health department or a private organization)?

__ Yes, *please specify:*

__ No

5. Currently CDC wastewater surveillance data are publicly available for SARS-CoV-2 and monkeypox virus. CDC plans to test for additional priority targets in the future. Which of the following targets would you find most useful for wastewater surveillance?

__ Influenza A *[Select all that apply]*

__ Influenza B

__ RSV

__ Adenovirus

__ Norovirus

__ Measles

__ West Nile Virus

__ Dengue

__ Bacterial pathogens (e.g., *Campylobacter jejuni*)

__ Parasitic pathogens (e.g., *Cyclospora cayetanensis*)

__ Fungal pathogens (e.g., *Candida auris*)

__ Antibiotic resistance genes (e.g., colistin, vancomycin, and tetracycline resistance)

__ Other, *please specify*:

6. In your practice, please indicate the usefulness of the following potential levels of reporting...

a. ...based on geographic location:

By city __ Very useful __ Somewhat useful __ Slightly useful

By county __ Very useful __ Somewhat useful __ Slightly useful

By state __ Very useful __ Somewhat useful __ Slightly useful

Nationwide __ Very useful __ Somewhat useful __ Slightly useful

b. ...based on type of congregate setting:

Hospitals __ Very useful __ Somewhat useful __ Slightly useful

Long-term care facilities __ Very useful __ Somewhat useful __ Slightly useful

Schools (K-12) __ Very useful __ Somewhat useful __ Slightly useful

Universities __ Very useful __ Somewhat useful __ Slightly useful

Jails/detention centers __ Very useful __ Somewhat useful __ Slightly useful

Homeless shelters __ Very useful __ Somewhat useful __ Slightly useful

Other reporting levels of interest specified:

7. How would you prefer to receive wastewater surveillance information? *[Select all that apply]*

__ Not applicable, do not wish to receive

__ Web site

__ App on a mobile device

__ Emailed reports

Comments / other suggestions:

8. Please provide any specific example(s) of how wastewater surveillance has affected/could affect your clinical practice.

9. Additional comments about wastewater surveillance are welcome:

Appendix Table 1. Differences in US. infectious disease physicians' responses to the Emerging Infections Network survey by US Census Bureau Region of residence, February-March 2024*,†,‡

Survey Responses	Northeast (N = 108) No. (%)	Midwest (N = 107) No. (%)	South (N = 126) No. (%)	West (N = 107) No. (%)	Pearson's chi-square p-value
**Wastewater surveillance conducted in county or state of work**					**<0.01‡**
No. respondents	n = 108	n = 107	n = 126	n = 107	
Yes	63 (58)	78 (73)	69 (55)	76 (71)	
No or Unsure	45 (42)	29 (27)	57 (45)	31 (29)	
**Awareness and frequency of review of wastewater surveillance data**					0.1
No. respondents	n = 107	n = 107	n = 126	n = 106	
Aware and review regularly	26 (24)	24 (22)	18 (14)	30 (28)	
Aware but do not review regularly	59 (55)	57 (53)	75 (60)	60 (57)	
Not aware of these data	22 (21)	26 (24)	33 (26)	16 (15)	
**Use CDC or non-CDC wastewater Web sites**					0.6
No. respondents	n = 108	n = 107	n = 126	n = 107	
Yes	59 (55)	54 (51)	58 (46)	55 (51)	
No	49 (45)	53 (50)	68 (54)	52 (49)	
**Microbial targets respondents chose as potentially useful for surveillance§**					
No. respondents	n = 108	n = 107	n = 126	n = 107	
Influenza A	75 (69)	70 (65)	83 (66)	74 (69)	0.9
Influenza B	57 (53)	63 (59)	75 (60)	59 (55)	0.7
RSV	58 (54)	57 (53)	66 (52)	66 (62)	0.5
Norovirus	62 (57)	58 (54)	67 (53)	58 (54)	0.9
Measles	60 (56)	58 (54)	69 (55)	58 (54)	1.0
Antibiotic Resistance	50 (46)	44 (41)	53 (42)	51 (48)	0.7
West Nile Virus	42 (39)	42 (39)	41 (33)	47 (44)	0.4
Fungal	36 (33)	32 (30)	39 (31)	31 (29)	0.9
Bacterial	32 (30)	28 (26)	30 (24)	27 (25)	0.8
Adenovirus	22 (20)	23 (22)	31 (25)	34 (32)	0.2
Parasitic	26 (24)	22 (21)	31 (25)	24 (22)	0.9
Dengue	22 (20)	20 (19)	37 (29)	21 (20)	0.2
	**No. / No. respondents (%)**	**No. / No. respondents (%)**	**No. / No. respondents (%)**	**No. / No. respondents (%)**	**Pearson's chi-square p-value**
**Reported geographic reporting level as very useful§,¶**					
City	72/ 103 (70)	63/ 98 (64)	78/ 121 (65)	68/ 102 (67)	0.8
County	64/ 104 (62)	61/ 101 (60)	84/ 123 (68)	68/ 103 (66)	0.6
State	32/ 104 (31)	19/ 100 (19)	28/ 123 (23)	24/ 99 (24)	0.3
Nation	15/ 101 (15)	14/ 96 (15)	20/ 120 (17)	15/ 101 (15)	1.0
**Reported facility reporting level as very useful§,¶**					
Hospital	65/ 103 (63)	56/ 100 (56)	74/ 121 (61)	59/ 102 (58)	0.7
Long-term care facility	64/ 102 (63)	51/ 100 (51)	62/ 120 (52)	53/ 101 (53)	0.3
K-12 school	43/ 102 (42)	29/ 96 (30)	48/ 119 (40)	31/ 97 (32)	0.2
University	31/ 100 (31)	23/ 96 (24)	39/ 118 (33)	28/ 94 (30)	0.5
Jail/detention center	31/ 101 (31)	25/ 97 (26)	38/ 118 (32)	35/ 98 (36)	0.5
Homeless shelter	42/ 99 (42)	24/ 96 (25)	32/ 116 (28)	40/ 99 (40)	**0.01‡**

Survey Responses	Northeast (N = 108) No. (%)	Midwest (N = 107) No. (%)	South (N = 126) No. (%)	West (N = 107) No. (%)	Pearson's chi-square p-value

*The Infections Disease Society of America (IDSA) Emerging Infections Network (EIN) is a provider-based emerging infections sentinel network established in 1995 to assist the US Centers for Disease Control and Prevention (CDC) and other public health authorities with surveillance for emerging infectious diseases and related phenomena. The electronic survey was distributed via three emails in February and March 2024 to all US infectious disease EIN physician members.
†Respondents who did not reply to specific question were excluded from analyses for that question.
‡Responses were statistically significantly different at the 0.05 level.
§At the time of the survey, the National Wastewater Surveillance System was reporting wastewater surveillance data publicly for SARS-CoV-2 and monkeypox virus at the sampling site, state, and national levels.
¶Respondents were asked to rate geographic and facility reporting levels for wastewater surveillance data as slightly, somewhat, or very useful.

Appendix Table 2. Differences in US infectious disease physicians' responses to the Emerging Infections Network survey by years of experience after infectious disease fellowship, February-March 2024[*,†,‡]

Survey Responses	<15 y (N = 195) No. (%)	≥15 y (N = 253) No. (%)	Pearson's chi-square p-value
**Wastewater surveillance conducted in county or state of work**			0.8
No. respondents	n = 195	n = 253	
Yes	123 (63)	163 (64)	
No or Unsure	72 (37)	90 (36)	
**Awareness and frequency of review of wastewater surveillance data**			0.2
No. respondents	n = 194	n = 252	
Aware and review regularly	36 (19)	62 (25)	
Aware but do not review regularly	111 (57)	140 (56)	
Not aware of these data	47 (24)	50 (20)	
**Use CDC or non-CDC wastewater Web sites**			0.09
No. respondents	n = 195	n = 253	
Yes	89 (46)	137 (54)	
No	106 (54)	116 (46)	
**Targets respondents chose as potentially useful for surveillance§**			
No. respondents	n = 195	n = 253	
Influenza A	137 (70)	165 (65)	0.3
Influenza B	118 (61)	136 (54)	0.2
RSV	117 (60)	130 (51)	0.09
Norovirus	105 (54)	140 (55)	0.8
Measles	118 (61)	127 (50)	**0.04‡**
Antibiotic Resistance	99 (51)	99 (39)	**0.02‡**
West Nile Virus	78 (40)	94 (37)	0.6
Fungal	63 (32)	75 (30)	0.6
Bacterial	52 (27)	65 (26)	0.9
Adenovirus	50 (26)	60 (24)	0.7
Parasitic	45 (23)	58 (23)	1.0
Dengue	44 (23)	56 (22)	1.0
	**No. / No. respondents (%)**	**No. / No. respondents (%)**	**Pearson's chi-square p-value**

Survey Responses	<15 y (N = 195) No. (%)	≥15 y (N = 253) No. (%)	Pearson's chi-square p-value
**Reported geographic reporting level as very useful§,¶**			
City	135/ 186 (73)	146/ 238 (61)	**0.02‡**
County	121/ 188 (64)	156/ 243 (64)	1.0
State	46/ 188 (25)	57/ 238 (24)	1.0
Nation	25/ 185 (14)	39/ 233 (17)	0.4
**Reported facility reporting level as very useful§,¶**			
Hospital	126/ 187 (67)	128/ 239 (54)	**<0.01‡**
Long-term care facility	109/ 187 (58)	121/ 236 (51)	0.2
K-12 school	69/ 182 (38)	82/ 232 (35)	0.7
University	57/ 178 (32)	64/ 230 (28)	0.4
Jail/detention center	59/ 182 (32)	70/ 232 (30)	0.7
Homeless shelter	67/ 182 (37)	71/ 228 (31)	0.3

*The Infections Disease Society of America (IDSA) Emerging Infections Network (EIN) is a provider-based emerging infections sentinel network established in 1995 to assist the US Centers for Disease Control and Prevention (CDC) and other public health authorities with surveillance for emerging infectious diseases and related phenomena. The electronic survey was distributed via three emails in February and March 2024 to all US infectious disease EIN physician members.

†Respondents who did not reply to specific question were excluded from analyses for that question.

‡Responses were statistically significantly different at the 0.05 level.

§At the time of the survey, the National Wastewater Surveillance System was reporting wastewater surveillance data publicly for SARS-CoV-2 and monkeypox virus at the sampling site, state, and national levels.

¶Respondents were asked to rate geographic and facility reporting levels for wastewater surveillance data as slightly, somewhat, or very useful.

Appendix Table 3. Differences in US infectious disease physicians' responses to the Emerging Infections Network survey by practice type, February-March 2024*,†,‡

Survey Responses	Adult (N = 356) No. (%)	Pediatric (N = 92) No. (%)	Pearson's chi-square p-value
**Wastewater surveillance conducted in county or state of work**			0.8
No. respondents	n = 356	n = 92	
Yes	229 (64)	57 (62)	
No or Unsure	127 (36)	35 (38)	
**Awareness and frequency of review of wastewater surveillance data**			0.7
No. respondents	n = 356	n = 90	
Aware and review regularly	81 (23)	17 (19)	
Aware but do not review regularly	197 (55)	54 (60)	
Not aware of these data	78 (22)	19 (21)	
**Use CDC or non-CDC wastewater Web sites**			0.8
No. respondents	n = 356	n = 92	
Yes	178 (50)	48 (52)	
No	178 (50)	44 (48)	
**Microbial targets respondents chose as potentially useful for surveillance§**			
No. respondents	n = 356	n = 92	
Influenza A	243 (68)	59 (64)	0.5
Influenza B	200 (56)	54 (59)	0.8

Survey Responses	Adult (N = 356) No. (%)	Pediatric (N = 92) No. (%)	Pearson's chi-square p-value
RSV	190 (53)	57 (62)	0.2
Norovirus	186 (52)	59 (64)	0.05
Measles	191 (54)	54 (59)	0.5
Antibiotic Resistance	159 (45)	39 (42)	0.8
West Nile Virus	134 (38)	38 (41)	0.6
Fungal	119 (33)	19 (21)	**0.03**‡
Bacterial	92 (26)	25 (27)	0.9
Adenovirus	73 (21)	37 (40)	**<0.01**‡
Parasitic	85 (24)	18 (20)	0.5
Dengue	75 (21)	25 (27)	0.3
	**No. / No. respondents (%)**	**No. / No. respondents (%)**	**Pearson's chi-square p-value**
**Reported geographic reporting level as very useful§,¶**			
City	222/ 336 (66)	59/ 88 (67)	1.0
County	212/ 341 (62)	65/ 90 (72)	0.1
State	75/ 337 (22)	28/ 89 (32)	0.1
Nation	49/ 331 (15)	15/ 87 (17)	0.7
**Reported facility reporting level as very useful§,¶**			
Hospital	202/ 337 (60)	52/ 89 (58)	0.9
Long-term care facility	198/ 336 (59)	32/ 87 (37)	**<0.01**‡
K-12 school	103/ 327 (32)	48/ 87 (55)	**<0.01**‡
University	88/ 323 (27)	33/ 85 (39)	0.05
Jail/detention center	111/ 328 (34)	18/ 86 (21)	**0.03**‡
Homeless shelter	117/ 324 (36)	21/ 86 (24)	0.06

*The Infections Disease Society of America (IDSA) Emerging Infections Network (EIN) is a provider-based emerging infections sentinel network established in 1995 to assist the US Centers for Disease Control and Prevention (CDC) and other public health authorities with surveillance for emerging infectious diseases and related phenomena. The electronic survey was distributed via three emails in February and March 2024 to all US infectious disease EIN physician members.

†Respondents who did not reply to specific question were excluded from analyses for that question.

‡Responses were statistically significantly different at the 0.05 level.

§At the time of the survey, the National Wastewater Surveillance System was reporting wastewater surveillance data publicly for SARS-CoV-2 and monkeypox virus at the sampling site, state, and national levels.

¶Respondents were asked to rate geographic and facility reporting levels for wastewater surveillance data as slightly, somewhat, or very useful.
